# Periodic detection and disinfection maintenance of dental unit waterlines in dental simulation head model laboratories

**DOI:** 10.1038/s41598-025-89010-3

**Published:** 2025-02-12

**Authors:** Biyao Li, Yuya Hu, Yiqi Wang, Chengqi Zhang, Zimo Wang, Xujie Peng, Jianying Feng

**Affiliations:** 1 School of Stomatology, Zhejiang Chinese Medical University, Zhejiang, China; 2 Department of Stomatology, School/Hospital of Stomatology, Zhejiang Chinese Medical University, Zhejiang Chinese Medical University, Zhejiang, China

**Keywords:** Dental simulation head model laboratory, Dental unit waterlines, Microbial contamination, Organochlorine disinfectant, Chlorine dioxide, High-throughput sequencing, Microbiology, Health care, Health occupations

## Abstract

Dental simulation head model laboratories are crucial for clinical simulation training for stomatological students, yet the maintenance of their dental unit waterlines (DUWLs) has been overlooked. This study investigated water contamination in DUWLs within these laboratories and proposed solutions. Water samples were collected from 12 dental chairs in three laboratories at three time points: the beginning, middle, and end of the semester. At the start of the semester, severe contamination was observed, with colony counts of 11,586$\:\pm\:$1715 CFU/ml for high-speed handpieces and 5375$\:\pm\:$874 CFU/ml for three ways syringes. As the semester progressed, colony counts gradually decreased but remained above clinical thresholds. Both 20 mg/L organochlorine disinfectant and 20 mg/L chlorine dioxide were effective in reducing bacterial contamination below standard ranges three days post-disinfection. Microbial diversity analysis revealed *Proteobacteria* and *Bacteroidota* as the dominant bacterial phyla, with *Ascomycota* as the dominant fungal phylum. Potentially pathogenic bacteria such as *Pseudomonas*, *Burkholderia-Caballeronia-Paraburkholderia*, *Ralstonia*, *Mycobacterium*, *Legionella*, *Paenibacillus*, *Streptomyces*, *Acinetobacter*, and *Prevotella*, as well as fungi like *Fusarium* and *Penicillium*, were detected. Therefore, urgent attention is needed to address DUWL contamination in dental laboratories, and it is recommended to disinfect DUWLs using either 20 mg/L organochlorine disinfectant or 20 mg/L chlorine dioxide every three days.

**Supplementary Information:**

The online version contains supplementary material available at 10.1038/s41598-025-89010-3.

## Background

Dental simulation head model laboratories constitute a vital role in stomatological education, with the primitive objective of cultivating the clinical proficiency of dental students. In the laboratory, students engage in the practice of various dental treatments such as tooth preparation, restoration and endodontic therapy on simulation heads, which utilize dental unit waterlines (DUWLs). DUWLs are composed of narrow and elongated interconnected tubes and linked to high-speed handpieces and three ways syringes. The combination of factors such as small tube diameters, high surface-to-volume ratios, rough surface, and slow water flow rates, facilitate the formation and residence of biofilms on the inner walls of the tubes^1,2^. Furthermore, debris, microorganisms, and chemical reagents generated during students’ practice may enter and deposit in the waterlines, leading to water quality deterioration and an increased risk of cross-infection, thereby affecting the health of both teachers and students.

Nevertheless, the disinfection and maintenance of DUWLs in dental simulation head model laboratories have been overlooked for an extended period. The majority of current research focuses on the DUWLs in clinical settings, including contamination levels, bacterial species, harm to patients, and sterilization management (disinfectant types, concentrations, and disinfection frequencies)^1,3^. Various regulations have been formulated to control clinical DUWLs contamination. In 1996, the American Dental Association (ADA) set a limit of ≤ 200 CFU/mL on the aerobic mesophilic heterotrophic bacteria in the output water of the dental unit^4^. In 2003, the US Centers for Disease Control and Prevention (CDC) recommended that the bacteria in dental unit water should not be less than 500 CFU/mL^5^. Moreover, in some EU countries, the drinking water standard of < 100 CFU/ml is referenced^6^. And in 2024, China also clarified in the standard for prevention and control of healthcare associated infection in dental outpatient department and clinic that the water from DUWLs should meet the drinking water standard (< 100 CFU/ml)^7^. The primary sources of contamination in clinics are bacteria from tap water and patients, in addition to the initial contamination of DUWLs among new and never used dental chairs^8^. Currently, medical regulatory authorities have established the testing of DUWLs in clinics as a standard regulatory requirement^9^. However, the contamination of DUWLs in dental simulation head model laboratories remains an overlooked area, with no pertinent reports to date.

This study analyzed the contamination level of DUWLs periodically through a semester-long investigation, and assessed the disinfection effects of two commonly used disinfectants (20 mg/L organochlorine disinfectant and 20 mg/L chlorine dioxide). Additionally, we utilized high-throughput sequencing technology to identify the microbial communities. This study seeks to elevate awareness regarding the serious contamination of DUWLs in dental simulation head model laboratories and proposes viable strategies for mitigating such contamination.

## Methods

### Study subjects

**Fig. 1:**
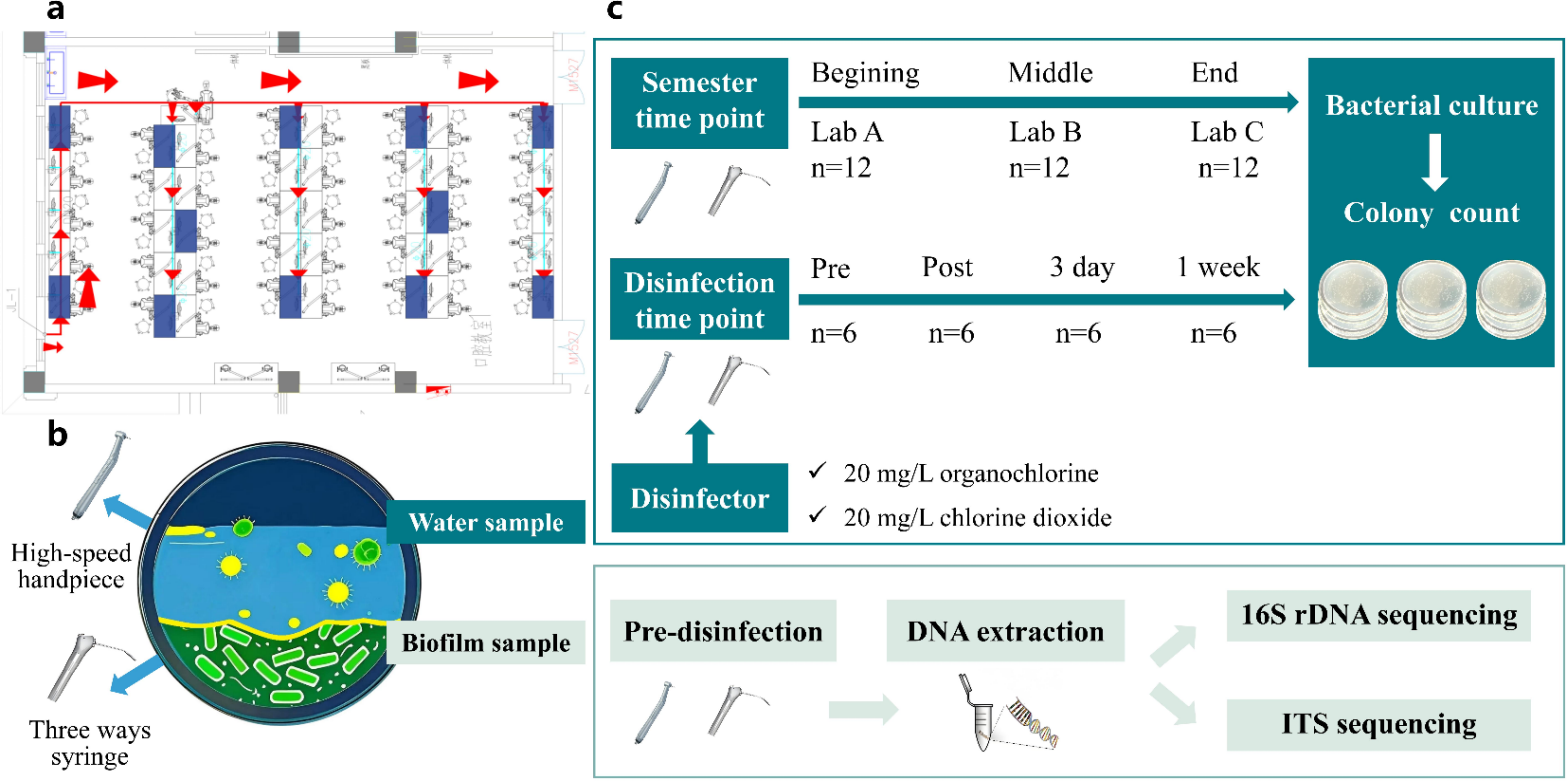
Study design. (**a**) shows the oral simulation head model laboratory waterway system. The chosen subjects are marked in blue. (**b**) depicts the cross-section of waterlines. (c) illustrates the experimental protocol for assessing water sample contamination levels and disinfection efficacy, as well as the procedure for performing biodiversity analysis on the biofilm formed within the water pipe.

Three oral simulation head model laboratories from the School of Stomatology, Zhejiang Chinese Medical University were selected, with each laboratory featuring identically arranged DUWLs (Fig. 1a). The three laboratories were simultaneously opened to three classes in the same grade during one semester, with consistent usage frequency and duration in order to minimize sample error among the three laboratories. All the laboratories had not undergone systematic DUWL disinfection or maintenance procedures before. For each laboratory we chose 12 simple dental chairs (SDCs) (NISSIN NS-1000, China) (marked in blue on Fig. 1a) equipped with oral treatment devices including high-speed handpieces and three ways syringes. The DUWLs in the three laboratories were immediately disinfected out of biosafety concerns after sampling. The municipal tap water used in the laboratories met the microbial standards outlined in the Chinese national standard (GB/T 5750.12–2006). Tap water tests were conducted both before and after the study, with the results confirming compliance with the Chinese national standard.

## Water sample collection

Water samples were collected from three laboratories at the beginning (Laboratory A), middle (Laboratory B), and end (Laboratory C) of the semester (Fig. 1). For each time point, water samples were collected from the waterlines of one high-speed handpiece and one three ways syringe on each dental chair, with two samples collected from each waterline as duplicates. For the three ways syringe waterline, we removed the replaceable jet heads of the syringe, pressed the water spray button for 30 s, then wiped and sterilized the connector with 75% ethanol and collected 10 ml of water from the syringe using a sterile sampling bottle. For the high-speed handpiece waterline, the handpiece was not installed, we stepped back the metal cap, then rinsed, wiped and sampled as described above. All water samples were stored in a 4 °C refrigerator within 30 min after collection and inoculated within 6 h.

## Disinfection

250 mg/tablet organic chlorine effervescent tablets (Health Essence, China) and 100 mg/tablet chlorine dioxide effervescent tablets (Basteur, China) were prepared respectively with purified water to form chlorine-containing disinfectants at a concentration of 20 mg/L, which were prepared and used immediately. The 12 SDCs in laboratory B were randomly divided into two groups, with one group disinfected using 20 mg/L organochlorine disinfectant and the other using 20 mg/L chlorine dioxide disinfectant. First, the dental instruments attached to the SDC were removed, 1 L of disinfectant was filled in a separate water reservoir, followed by continuous drainage for 2 min, static placement for 20 min, and finally rinsing with 1 L of tap water. Water samples were collected before disinfection, immediately after disinfection, and 3 and 7 days after disinfection.

## Bacterial culture

100 µl of each water sample was inoculated onto a nutrient agar plate. The plates were incubated at 37 °C for 48 h in an incubator. Viable bacterial counts were calculated as CFU/ml, and a colony count exceeding 100 CFU/ml was considered contaminated.

### Statistical analysis

Statistical analysis was carried out using SPSS 24.0. The Shapiro-Wilk test was used to assess the normality of the experimental data, and the F-test with *P* > 0.05 was used to verify the homogeneity of variances. Independent T-tests were conducted to analyze the contamination levels of the high-speed handpiece and three ways syringe waterlines at different time points. The contamination rate was calculated as follows:

$${\text{Contamination}}\;{\text{Rate}} = \:\frac{{{\text{Number}}\:{\text{of}}\:{\text{contaminated}}\:{\text{samples}}}}{{{\text{Total}}\:{\text{number}}\:{\text{of}}\:{\text{samples}}}} \times \:100\%$$

Paired T-tests were used to statistically analyze the bacterial colony counts in water samples before and after disinfection to assess the disinfection effect. Independent T-tests were used to analyze the contamination levels of the high-speed handpiece and three ways syringe waterlines. A P-value < 0.05 was considered statistically significant.

### Biofilm sample collection

Eight biofilm samples were randomly obtained from the water pipes of four SDCs equipped with high-speed handpieces (Group 1) and three ways syringes (Group 2) in Laboratory A at the beginning of the study. Prior to sample collection, the water pipes underwent a 30-second flushing procedure and were sterilized using 75% ethanol, then detached from the control device. A sterile scissor was used to excise and discard a 5-millimeter segment from the tube’s anterior end. An aseptic nasal swab, measuring 2 mm in diameter and 17 millimeters in length, was inserted into the tube, rotating and traversing for 30 s to maximize biofilm collection. The swab’s head was subsequently preserved in an EP tube. All EP tubes were promptly stored at -80 °C within a thirty-minute timeframe to ensure sample integrity.

## DNA extraction, 16 S rDNA and ITS gene sequencing and data analysis

Biofilm samples were centrifuged at 8000 g for 3 minutes with ddH2O, and the sediment was collected. DNA was extracted using the CTAB kit (Hangzhou Zeheng Biotechnology, China) following the manufacturer’s instructions. PCR amplification was performed with primers targeting the V3-V4 hypervariable regions of bacterial 16S rRNA genes (341F: 5’-CCTAYGGGRBGCASCAG-3’, 806R: 5’-GGACTACNNGGGTATCTAAT-3’) and the fungal ITS genes (ITS1F: 5’-CTTGGTCATTTAGAGGAAGTAA − 3’, ITS2R: 5’-GCTGCGTTCTTCATCGATGC-3’). The amplicon pools were prepared for sequencing using the Illumina NovaSeq 6000 platform (Shanghai Wei Huan Biological Technology, Shanghai, China). The sequences were analyzed employing Qiime 2.

## Results

### Current status of DUWLs contamination

**Fig. 2:**
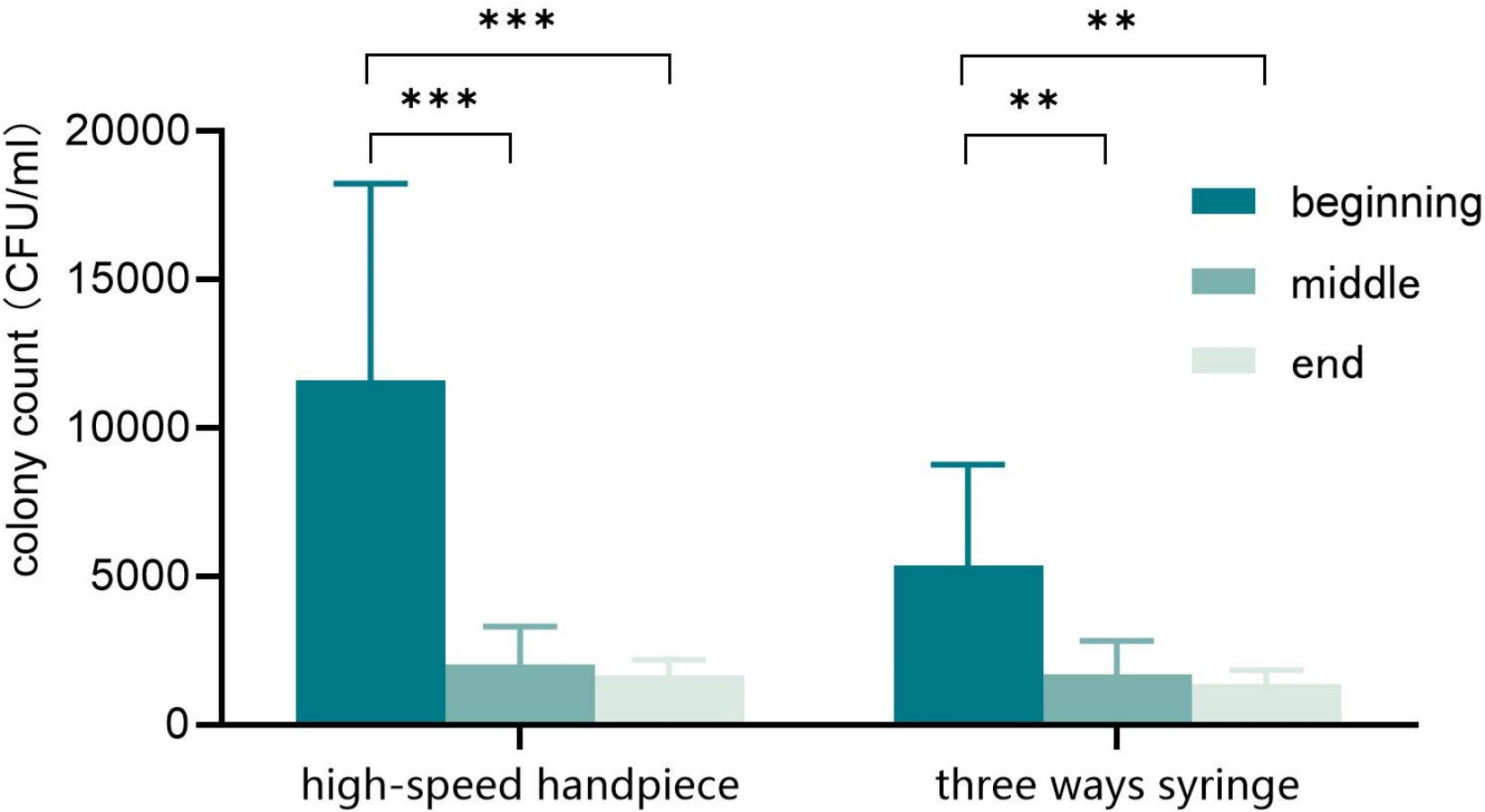
Colony count of high-speed handpiece and three ways syringe output water during one semester. Independent T-tests were conducted. *P < 0.05, **P < 0.01, ***P < 0.001.

The bacterial colony counts and contamination rates in the waterlines of high-speed handpieces and three ways syringes at different time points are shown in Table 1; Fig. 2. At the beginning, middle, and end of the semester, the bacterial colony counts in the waterlines of both high-speed handpieces and three ways syringes exceeded the standard of 100 CFU/ml, indicating a high level of contamination with a contamination rate of 100%. As students used the equipment and the waterlines were flushed more frequently, the bacterial colony counts in the waterlines of both high-speed handpieces and three ways syringes significantly decreased during the middle and end of the semester.

**Table 1:** High-speed handpiece and three ways syringe output water contamination at different period of the semester.

Time point	Colony count (CFU/ml)^a^		T value	*P* value	Contamination rate (%)	
	High-speed handpiece	Three ways syringe			High-speed handpiece	Three ways syringe
Beginning	11,586$\:\pm\:$1715	5375$\:\pm\:$874	3.954	0.001	100.00	100.00
Middle	2041$\:\pm\:$327***	1690$\:\pm\:$295**	0.866	0.401	100.00	100.00
End	1680$\:\pm\:$131***	1372$\:\pm\:$119**	1.587	0.135	100.00	100.00

^a^ Mean ± standard deviation. Independent T-tests were conducted, vertically (to compare among different periods) and laterally (to compare between high-speed handpieces and three ways syringes). * Compared with the beginning, *P < 0.05, **P < 0.01, ***P < 0.001.

### Disinfection

Our previous study has demonstrated that 20 mg/L organochlorine disinfectant and 20 mg/L chlorine dioxide are effective in inactivating *Escherichia coli* and *Staphylococcus aureus*^10^. Therefore, these two disinfectants were selected in this study for the disinfection of DUWLs in dental laboratories.

**Fig. 3:**
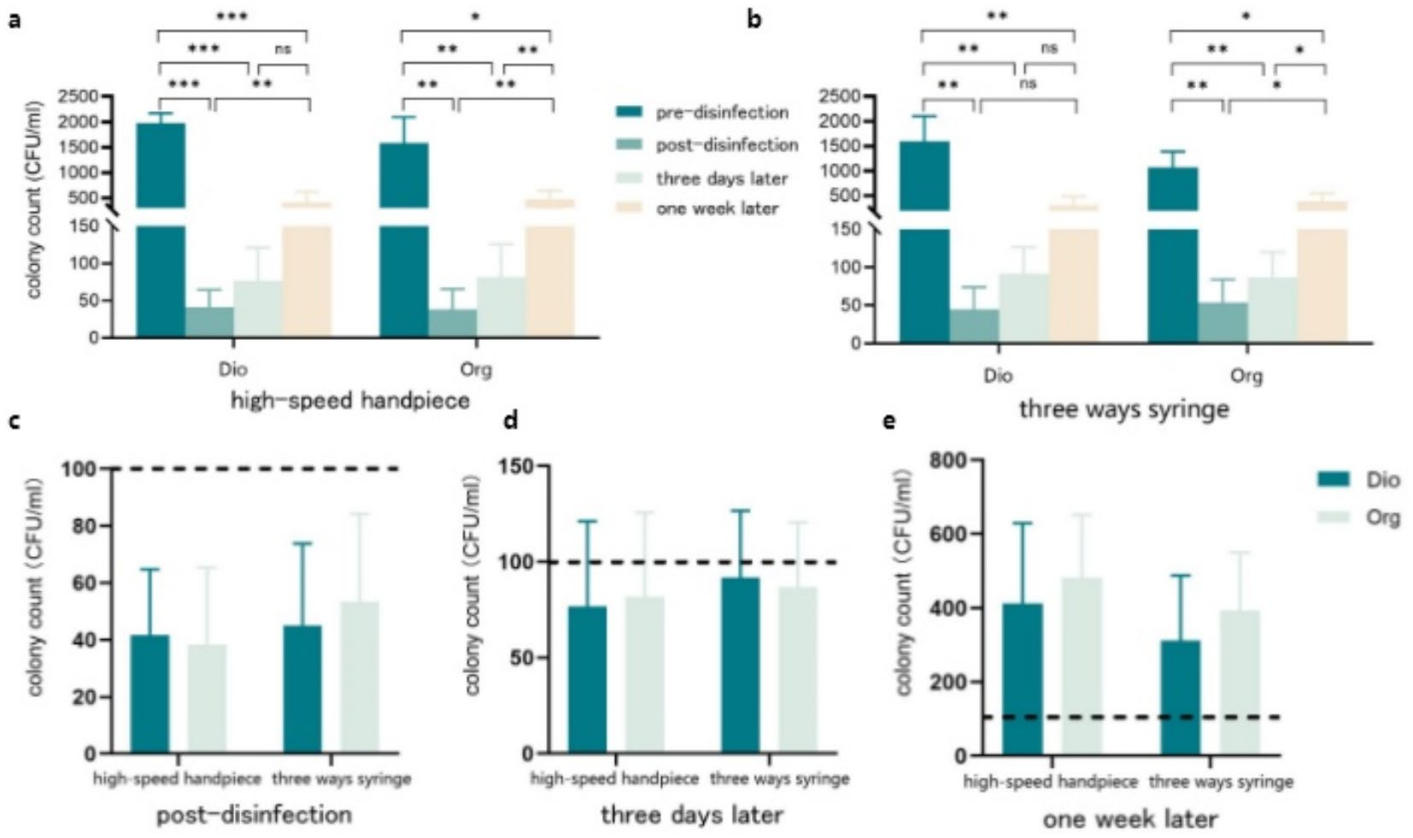
Colony counts before and after disinfection of chlorine dioxide and organochlorine disinfectants. (**a**) and (**b**) show the colony counts in the waterlines before and after disinfection for high-speed handpieces and three ways syringes, respectively. (**c**) (**d**) and (**e**) show the colony counts immediately post-disinfection, three days post-disinfection and one week post-disinfection, respectively. The dotted line stands the contamination line, over which indicates contamination. Dio and org are chlorine dioxide disinfectant and organochlorine disinfectant, respectively. Paired T-tests were conducted. *P<0.05, **P<0.01, ***P<0.001.

The results indicated that both disinfectants exhibited good bactericidal efficacy (Table 2; Fig. 3), with a significant reduction in bacterial colony counts in the waterlines after disinfection (*P* < 0.05), and the contamination rate dropped to 0%. Until one week post-disinfection, the colony counts remained significantly lower than those before disinfection (*P* < 0.05). However, compared to the immediate post-disinfection, the colony counts had increased significantly at this time point (*P* < 0.05), with the contamination rate rising to 100%, suggesting that a weekly disinfection frequency is insufficient to meet the requirements for colony control. Moreover, the average colony count remained below 100 CFU/ml three days post-disinfection, with approximately one-third of the waterlines contaminated. Additionally, there was no statistically significant difference in the bactericidal effect between the two disinfectants (*P* > 0.05).

**Table 2:** Colony counts and contaminated rate before and after treatment with two disinfectants.

Group	Time point	Colony count (CFU/ml)^a^		T value	P value	Contamination rate (%)	
		High-speed handpiece	Three ways syringe			High-speed handpiece	Three ways syringe
Dio	Pre-disinfection	1978$\:\pm\:$192	1595$\:\pm\:$510	1.521	0.189	100.00	100.00
	Post-disinfection	42$\:\pm\:$23^b^	45$\:\pm\:$29^b^	-0.213	0.840	0	0
	Three days later	77 ± 45^b^	92 ± 35^b^	-0.488	0.646	16.67	33.33
	One week later	412$\:\pm\:$217^bc^	312$\:\pm\:$176^b^	0.953	0.384	100.00	100.00
Org	Pre-disinfection	1577$\:\pm\:$517	1073$\:\pm\:$315	1.613	0.168	100.00	100.00
	Post-disinfection	38$\:\pm\:$27^b^	53$\:\pm\:$31^b^	-1.031	0.350	0	0
	Three days later	82 ± 44^b^	87 ± 34^b^	0.182	0.863	33.33	33.33
	One week later	482$\:\pm\:$170^bc^	393$\:\pm\:$157^bc^	0.750	0.487	100.00	100.00

^a^ Mean ± standard deviation. Paired T-tests were conducted vertically (to compare among different time points). Independent T-tests were conducted laterally (to compare between high-speed handpieces and three ways syringes). ^b^ Compared with pre-disinfection, *P* < 0.05. ^c^ Compared with post-disinfection, *P* < 0.05.

Dio and org are chlorine dioxide disinfectant and organochlorine disinfectant, respectively.

### Microbial community composition of the laboratory DUWL biofilm

**Fig. 4:**
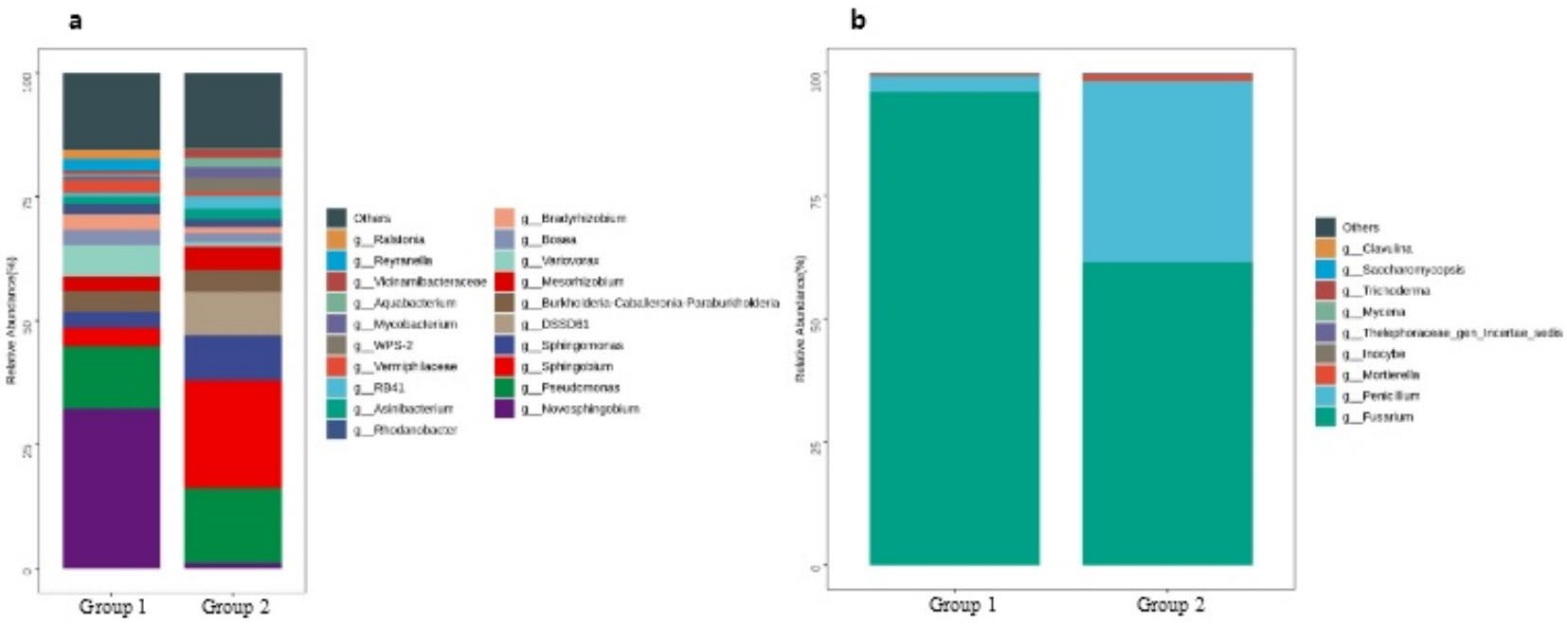
Relative abundance of bacterial (**a**) and fungal (**b**) community at genus level. Group 1 denotes samples from waterlines of high-speed handpieces and Group 2 denotes samples from waterlines of three ways syringes.

The main phyla of bacteria were *Proteobacteria* (63.17% in Group 1, 59.35% in Group 2) and *Bacteroidota* (29.04% in Group 1, 31.44% in Group 2) and that of fungi was *Ascomycota* (99.45% in Group 1, 98.24% in Group 2) in all samples. At the class level, *Alphaproteobacteria* (43.41% in Group 1, 39.53% in Group 2), *Bacteroidia* (29.04% in Group 1, 31.44% in Group 2), and *Gammaproteobacteria* (19.75% in Group 1, 19.82% in Group 2) were the dominant classes of bacteria, and *Sordariomycetes* (96.08% in Group 1, 61.44% in Group 2) and *Eurotiomycetes* (3.36% in Group 1, 36.80% in Group 2) were the dominant classes of fungi. The most abundant genera of bacteria were *Novosphingobium* (32.25%) and *Pseudomonas* (12.59%) in Group 1, and *Sphingobium* (21.77%) and *Pseudomonas* (15.09%) in Group 2. The most abundant genera of fungi were *Fusarium* (96.01% in Group 1, 61.46% in Group 2) and *Penicillium* (3.36% in Group 1, 36.81% in Group 2). The relative abundance of microbial community composition at genus level is illustrated in Fig. 4.

Potential human pathogens detected in biofilm samples comprised 9 bacterial genera and 2 fungal genera. Specifically, the bacterial genera with a relative abundance over 1% included *Pseudomonas* (12.59% in Group 1, 15.09% in Group 2), *Burkholderia-Caballeronia-Paraburkholderia* (4.11% in Group 1, 4.45% in Group 2), *Mycobacterium* (2.14% in Group 2), and *Ralstonia* (1.79% in Group 1). Other pathogenic bacterial genera were *Legionella*, *Paenibacillus*, *Streptomyces*, *Acinetobacter*, and *Prevotella*. Notably, the fungal genera *Fusarium* and *Penicillium* were identified as the most abundant and potentially pathogenic to humans in both groups.

## Discussion

In 1963, Blake first discovered the presence of a significant number of bacteria in DUWLs and raised the concern of cross-infection risks^11^. Since then, DUWL contamination has gradually garnered attention in oral clinical management. A survey in Switzerland found that 70% of DUWLs were contaminated with a high number of aerobic mesophilic heterotrophic bacteria and 90% of dental offices had a medium level of air contamination^12^. The primary reasons for DUWL contamination include: (1) bacteria inherently present in the water supply system^13^; (2) back-suction of dental handpieces^14^; (3) biofilm formation within the waterlines^15^; (4) water stagnation due to narrow lumens, slow water flow, and the intermittent use patterns^15–18^. Studies have shown that bacteria in DUWLs mainly consist of bacteria unique to the water supply system, opportunistic pathogens, and oral flora, including *Staphylococcus*, *Streptococcus*, *Actinomyces*, and *Legionella pneumophila*^19–21^. Additionally, fungi (such as *Candida albicans*), viruses, and parasites have also been found^1,22–24^. These microorganisms not only contaminate the water quality at the outlet but may also appear in aerosols generated during the operation of dental instruments, thereby increasing the risk of cross-infection, especially opportunistic infections^25^. In 2012, Lancet reported a case of an 82-year-old patient who died from *Legionella* infection, with a medical history revealing only two dental treatments and no other evident exposure risks^18^. In 2016, 71 children were diagnosed with odontogenic *nontuberculous mycobacterial* infections after receiving endodontic treatment^26^.

However, contamination in DUWLs within dental laboratories has been overlooked for an extended period. This study represents the first to focus on contamination in the waterlines of SDCs in oral simulation head model laboratories. The findings reveal that the average colony count in unsterilized DUWLs amounts to 1.2 × 10^4^ CFU/ml, significantly exceeding the domestic water quality standard of 100 CFU/ml. Existing research has demonstrated that, in clinical settings, colony counts in DUWLs can reach as high as 1.4 × 10^5^ and 1.8 × 10^6^ CFU/ml^27,28^. Notably, the colony count in the laboratory DUWLs examined in this study was slightly lower than those observed in clinical environments, potentially due to the use of resin teeth or autoclaved isolated teeth for student practice, thereby avoiding contamination from aspirated patient saliva, as typically encountered in clinical practices^14,29^. The contamination rate was 100% in three laboratories at the beginning, middle, and end of the semester, which may be attributed to the configuration of the laboratory waterline system, where 40 SDC waterlines are interconnected in series, thereby enhancing the likelihood of contamination throughout the entire laboratory. Furthermore, the average colony count detected in high-speed handpiece waterlines was generally higher than that in three ways syringes at various time points, indicating that the back-suction effect of high-speed handpieces significantly contributed to waterline contamination. Additionally, a correlation was identified between the degree of contamination in SDC waterlines and their frequency of use; specifically, the highest colony counts in high-speed handpiece and three-way syringe waterlines were observed at the beginning of the semester, following a downtime in holiday. As usage frequency increased, the contamination level decreased during the middle and end of the semester, yet remained at a high level overall. These findings suggest that DUWLs in laboratories are prone to the accumulation of various dirt and microorganisms during use, with bacteria readily proliferating within the pipes and forming biofilms in the downtime that are difficult to eliminate, thus posing potential sources of infection^30^. Based on the aforementioned rationale, CDC recommends rinsing as a physical means of reducing contamination during clinical procedures, with each patient undergoing rinsing for 20–30 s post-treatment^5^. However, rinsing alone is insufficient. A study has demonstrated that while rinsing significantly reduces colony counts in the output water, it still fails to meet the acceptable standard^31^, which is similar to the results of our study. Consequently, regular disinfection and maintenance of DUWLs are imperative.

The utilization of chemical disinfectants for the cleansing of DUWLs is considered the most expedient and efficacious approach to manage contamination, encompassing hydrogen peroxide, chlorine-containing disinfectants, slightly acidic electrolyzed water, etc^32–35^. Illustratively, Ditommaso et al. found that hydrogen peroxide (Peroxy Ag^+^) at 600 ppm could rapidly kill 99.99% of *Legionella* in DUWLs^36^; Agahi et al. sanitized DUWLs weekly using 0.2% chlorhexidine and found that colony counts in water samples decreased to less than 200 CFU/ml after four weeks^37^. Chlorine dioxide and organochlorine disinfectants are frequently employed in dental clinics owing to their superior bactericidal efficacy, good biosafety, operational simplicity and controllable costs^1^. The organochlorine disinfectant, with trichloroisocyanuric acid (TCCA) as its primary constituent, has been proven to be a safe and effective alternative to free chlorine for the disinfection of drinking water^38^. TCCA, in solid form, is easy to handle and highly stable^38^. Our preceding study has proved that relative to 20 ml/L organochlorine disinfectants, 20 ml/L chlorine dioxide exhibits enhanced antimicrobial efficacy while inducing minimal cytotoxicity and minimal corrosion to equipment, thereby rendering it a safer option for sustained utilization^10^. Furthermore, it is less prone to generating harmful disinfection by-products such as chlorite and chlorate^10^. In terms of biofilm control, chlorine dioxide has exhibited efficacy due to its non-reactivity with most biofilm constituents, which facilitates penetration through the biofilm matrix and enables the inactivation of the bacteria harbored within^39^. Additionally, TCCA can rapidly establish an equilibrium with high chlorine content upon dissolution in water, rendering it a promising candidate for the control of planktonic growth and the cultivability of biofilms^38^. To verify the disinfection efficacy of both in laboratory DUWLs, we employed the 20 mg/L of organochlorine disinfectant and the 20 mg/L of chlorine dioxide, respectively, for DUWL disinfection. The results showed that both disinfectants were capable of effectively eradicating bacteria in the waterlines. Especially three days post-disinfection, the colony counts sustained below 100 CFU/ml. Therefore, in contrast to the daily disinfection requirement for DUWLs in clinical settings, we propose that disinfection of DUWLs in oral simulation head model laboratories should be conducted every three days. Furthermore, we suggest the implementation of rigorous “shock treatments” in the beginning and the end of each semester, aimed at minimizing the growth of biofilms and eradicating any biofilms formed during the downtime^30^.

Furthermore, we assessed the microbiota diversity of biofilm in DUWLs of high-speed handpieces and three ways syringes in oral simulation head model laboratories. In our study, the dominant bacteria at the phylum level was *Proteobacteria*, representing over 60% of the total sequences, and the abundant fungi was *Ascomycota* at the phylum level, with *Sordariomycetes* and *Eurotiomycetes* being prevalent at the class level. These findings align with previous reports on DUWLs in clinics^40,41,42^. The primary bacteria genera detected in our study were *Novosphingobium*, *Pseudomonas* and *Sphingobium*, which have also been reported as predominant in clinical settings, albeit with variations in the ratios and diversity of micro-organisms observed across studies^40–44^. The majority of bacteria detected in DUWLs of oral teaching laboratories were heterotrophic, belonging to common environmental, soil-associated, and aquatic species, with low pathogenicity. When comparing the bacterial species present in the waterlines of high-speed handpieces and three-way syringes in our laboratory settings, we observed minimal differences, with one notable exception: the polycyclic aromatic hydrocarbon-degrading bacterium, *Novosphingobium*. This bacterium was abundant in the waterlines of high-speed handpieces (32%), whereas it was scarcely detected in those of three ways syringes (1.10%), and it is an aquatic heterotroph. However, in clinical settings, where devices are exposed to the oral cavity, the microbial composition in DUWLs becomes more complex^1^. Therefore, it may be of greater significance to detect and discuss the differences in bacterial contamination between the waterlines of high-speed handpieces and three ways syringes in clinical practice. Nevertheless, there is currently a scarcity of research addressing the distinct bacterial species present in the waterlines of clinical three ways syringes and high-speed handpieces, as well as the specific reasons for these differences.

Some opportunistic pathogens that can cause disease in immunocompromised people have also been identified in DUWLs. In our study, opportunistic pathogens accounted for approximately 20% of the total bacteria genera in terms of relative abundance, which was close to clinical findings; the opportunistic pathogens found in common with clinical DUWLs were *Pseudomonas*, *Burkholderia-Caballeronia-Paraburkholderia*, *Ralstonia*, *Mycobacterium*, *Legionella*, *Acinetobacter* and *Prevotella*^41,42,44^. These opportunistic pathogens like *Legionella* and *Pseudomonas* can contaminate surrounding air and surfaces, and cause various infection^45^. *Legionella* primarily causes Legionnaires’ disease with fever, chills, chest pain, and respiratory distress^46^, while *Pseudomonas* can lead to localized oral infections and, in immunocompromised individuals, systemic infections like bacteremia^46^. Additionally, the abundant fungi at the genus level in our study were *Fusarium* and *Penicillium*, both of which have potential pathogenic properties. *Penicillium*, a common genus found in aquatic environmental studies, is known to cause respiratory diseases such as allergic rhinitis as an allergen agent^47^. Conversely, few studies have reported *Fusarium* as the dominant fungi in DUWL biofilm. *Fusarium* can cause both systemic infections and local infections, such as keratitis, and it can be disseminated through hand-eye contact and aerosols^48^, posing a significant risk in oral simulation head model laboratories where students may lack adequate precautions. Students may be infected through inhalation of aerosols, direct contact with contaminated water, or indirect contact with contaminated equipment during training.

Notably, the configurations and operational patterns of oral simulation head model laboratories may exacerbate the risk of infection due to aerosols and splatter, which harbor microbes from DUWLs^49^, such as *Legionella*, *Pseudomonas*, *Fusarium* and *Penicillium*, as aforementioned. A study simulating dental procedures and evaluating distribution of aerosols and splatter concluded that the highest contamination was confined to 1.0–1.5 m from the source, with a maximum dissemination distance reaching 4 m^50^. And aerosols below 15 μm can persist in the air for an average of 7.13 min and can travel an average distance of 25.45 m from their source^51^. Considering the aforementioned layout of our laboratory, with adjacent SDCs separated by approximately 1.2 m, this distance is significantly less than the clinically recommended spacing requirements for SDCs, which advocate for a minimum distance of 2.4 m in the absence of physical barriers^7^. Furthermore, during class, the 40 SDCs in the laboratory are in operation simultaneously, with a practice duration that far exceeds that of actual clinical treatments. Hence, the dissemination of aerosols and splatter in laboratories is substantially more severe than that in clinical settings, leading to a marked increase in the risk of laboratory infection. These findings underscore the potential adverse effects of DUWLs in oral laboratories, even in a non-diagnostic environment, and emphasize the importance of regular control of microbial contamination in laboratory DUWLs.

To the best of our knowledge, our study represents the pioneering endeavor to concentrate on the contamination of DUWLs in oral education settings. While offering valuable insights, this study has its limitations. Firstly, this study only enrolled three oral simulation head model laboratories at a single university, potentially hindering the generalizability of the results to all dental laboratories. Secondly, the assessment of disinfection efficacy relied solely on the reduction in colony counts, without delving into the composition of the colonies post-disinfection. Lastly, the investigation into the interactions among microorganisms within biofilms, their pathogenicity, and antibiotic resistance may not have been sufficiently in-depth. Nevertheless, this study introduces a novel perspective and draws attention to the critical issue of contamination control in dental simulation head model laboratories.

## Conclusion

In conclusion, our findings indicate that contamination of DUWLs in oral simulation head model laboratories is also a cause for concern, particularly following extended periods of inactivity (such as at the beginning of a semester). The microbial community composition is similar to the previous reports of DUWLs in clinical settings. 9 bacterial genera and 2 fungal genera of potential human pathogens are detected in the biofilm samples, posing a potential risk of infection through hand-eye contact and aerosol transmission. The average bacterial count in DUWLs remained below 100 CFU/ml within three days post-disinfection with 20 mg/L of organochlorine or chlorine dioxide disinfectant. Consequently, we recommend that managers of oral laboratories implement regular disinfection using 20 mg/L of organochlorine disinfectant or 20 mg/L of chlorine dioxide every three days, and that both teachers and students should adopt adequate protective measures to mitigate the risk of infections in the laboratory.

## Electronic supplementary material

Below is the link to the electronic supplementary material.

Supplementary Material 1

## Data Availability

The datasets generated and analysed during the current study are available in the GSA repository (Accession number CRA020991, https://bigd.big.ac.cn/gsa/browse/CRA020991).

## Abbreviations

SDC: Simple dental chair
DUWL: Dental unit waterline
ADA: American Dental Association
CDC: Centers for Disease Control and Prevention
TCCA: Trichloroisocyanuric acid
