# Supplementary material for: Periodic detection and disinfection maintenance of dental unit waterlines in dental simulation head model laboratories
Source: Sci Rep. 2025 Feb 12;15:5234. doi: 10.1038/s41598-025-89010-3 (PMC11821815; doi:10.1038/s41598-025-89010-3)
Supplement: Supplementary file 1 — Supplementary Material 1 [file 41598_2025_89010_MOESM1_ESM.docx]

**Supplementary figure** Bacterial culture results of high-speed handpiece and three ways syringe output water during one semester. It qualitatively shows the concentration of colonies at different stages.


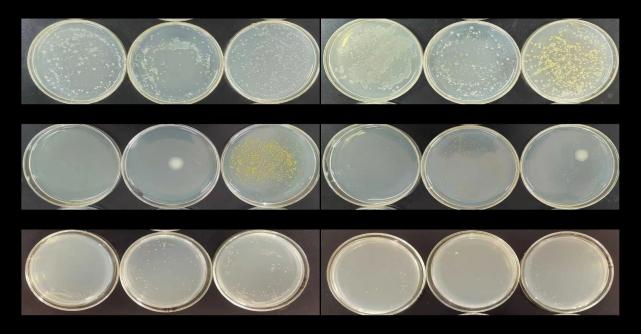

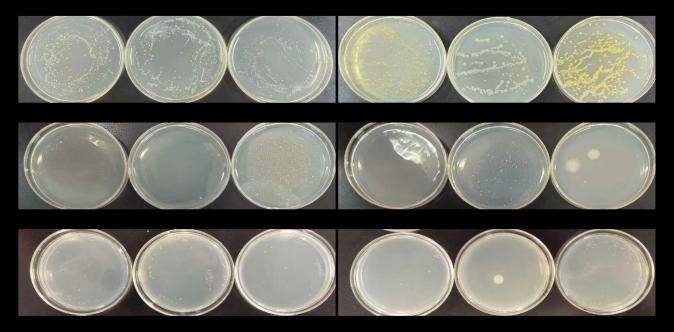


beginning

middle

end

high-speed handpiece

three ways syringe
